# Developing a melanoma-skin-on-a-chip integrated with vasculature using an edgeless skin reconstruction approach

**DOI:** 10.21203/rs.3.rs-10045259/v1

**Published:** 2026-06-29

**Authors:** Felikss Rumnieks, Deniz Ornek, Rolando Perez-Lorenzo, Hasan Erbil Abaci

**Affiliations:** Columbia University Irving Medical Centre, Department of Dermatology, NY, USA; National Institute of Research and Innovation,, Riga, Latvia; University of Latvia, Riga, Latvia; Columbia University, Department of Biomedical Engineering; Columbia University Irving Medical Centre, Department of Dermatology, NY, USA; Columbia University Irving Medical Centre, Department of Dermatology, NY, USA; Columbia University, Department of Biomedical Engineering

**Keywords:** human skin constructs, edgeless skin, melanoma modeling, tumor vascularization, tumor microenvironment

## Abstract

Melanoma is the most aggressive form of skin cancer, characterized by high metastatic potential and frequent resistance to targeted therapies. Traditional 2D cultures and animal models often fail to accurately mimic the complexity of the human tumor microenvironment (TME). To improve the recapitulation of the cellular and mechanical microenvironmental cues, we bioengineered a melanoma-skin-on-a-chip platform using an edgeless 3D skin reconstruction strategy, which allows for creating mechanically relevant skin microenvironments. The platform integrates a dermis compartment made of primary dermal fibroblasts, human umbilical vein endothelial cells (HUVECs), and melanoma spheroids (generated using MeWo and A375 cell lines), and an epidermis compartment made of differentiated layers of primary keratinocytes. Melanoma spheroids consist of a mixture of melanoma cells, fibroblasts and endothelial cells in collagen droplets, and when incorporated into the engineered dermis, form morphologically heterogeneous structures that mimicked *in vivo* tumor masses and late vertical progression phase of melanoma. The model successfully recapitulated key aspects of the tumor microenvironment, including increased peritumoral cancer-associated fibroblast (CAF) marker expression and extracellular matrix (ECM) remodeling. To evaluate the therapeutic relevance of the model, we evaluated the effects of MEK and BRF inhibitors on tumor cell proliferation and apoptosis. By day 7 post-tumor integration, vascular organization differed between melanoma backgrounds, with the MeWo construct showing 2.3-fold higher vessel area fraction and 3.65-fold higher vascular length density than the A375 construct, indicating enhanced vascular coverage and network density. This vascularized melanoma-skin-on-a-chip provides a human-relevant platform for studying melanoma invasion, response to therapeutics, and tumor-microenvironment dynamics. It holds strong potential for preclinical drug screening and offers a foundation for developing personalized therapeutic strategies.

## Introduction

Malignant melanoma, although the rarest major form of skin cancer, remains the most lethal due to its high metastatic potential. Its incidence continues to rise, making melanoma a growing public health concern globally. The malignant transformation of melanocytes primarily through UV-induced DNA damage, including double-strand breaks, leads to the accumulation of oncogenic mutations that promote uncontrolled proliferation, and invasion into the dermis. Current treatment strategies include surgical excision for early-stage disease and immunotherapy or targeted inhibition of the MAPK pathway (e.g., BRAF/MEK inhibitors) for metastatic disease. Although these approaches have significantly improved the outcome, intrinsic and acquired resistance remain common, and long-term survival for advanced disease remains limited; the 5-year relative survival is ~ 60% for regional (stage III) disease and ~ 16% for distant (stage IV) disease^1^.

These clinical challenges highlight the need for improved preclinical models to support melanoma drug development. However, translational success from animal studies to human trials remains poor, with fewer than 8% of oncology drug candidates demonstrating clinical efficacy^2–4^. This limitation is particularly pronounced in melanoma, where major differences between human and rodent skin, such as, epidermal thickness, immune composition, extracellular matrix (ECM) organization, and mechanical properties compromise predictive power^5,6^. As a result, melanoma drug discovery remains expensive, slow, and inefficient. Conventional in vitro approaches such as 2D cultures and tumor spheroids enable high-throughput screening and mechanistic studies but fail to reproduce the structural and mechanical complexity of native skin^7^.

To overcome these limitations, organotypic three-dimensional (3D) human melanoma-in-skin models have been developed, embedding melanoma cells or spheroids within reconstructed dermal-epidermal tissues. Compared to less complex *in vitro* alternatives, these systems better capture tumor-stroma interactions, invasion dynamics, and context-dependent drug responses. Such models have also enabled the investigation of early immune and vascular interactions during melanoma progression^8,9^.

Despite these advances, most existing melanoma skin models are engineered as 3D constructs with open boundaries, “edges”, on all sides, unlike the human skin, which is a continuous organ with no lateral open edges. Emerging evidence from our group and others^10,11^ indicates that such boundary conditions significantly influence cell-ECM dynamics, ECM deposition, tissue tension, and mechanical properties. Our recent work demonstrated that engineering the skin as a continuous, “edgeless” organ promotes physiological dermal architecture, improved collagen organization, and enhanced mechanical properties^12^ and provides a mechanomimetic microenvironment for disease modeling^13^. Given the strong dependence of melanoma invasion and plasticity on ECM structure and mechanics, these considerations are particularly relevant for modeling disease progression and therapeutic response^14^.

Here we present an edgeless human skin-melanoma chip (eSM-chip), capable of modeling melanoma progression and drug response in a mechanically relevant microenvironment. Using two established melanoma cell lines, A375 and MeWo, within a fibroblast-populated collagen dermis and a stratified epidermis, the eSMChip exhibits enhanced ECM remodeling and stromal activation, reflected by cancer-associated fibroblast (CAF) presence in proximity to the tumor. Treatment with BRAF inhibitor for A375 and MEK inhibitor for MeWo tumors reduces melanoma cell proliferation, confirming the translational relevance of the platform. Further incorporation of HUVECs enables vascular network formation within the engineered skin, capturing tumor-vascular crosstalk in a biomimetic environment and revealing differences in angiogenic capacity between cell lines. Together, the eSM-chip represents an advanced skin model of melanoma progression in a human-relevant context.

## Materials and Methods

### Cell culture

The human melanoma stable cell lines A375 (CRL-1619) and MeWo (HTB-65) were purchased from ATCC. Neonatal fibroblasts and keratinocytes were isolated from foreskins donated to the Presbyterian Hospital (Columbia University Institutional Review Board protocol AAAB2666). Fibroblasts and melanoma cell lines were cultured in Dulbecco’s modified Eagle’s medium (DMEM) with GlutaMAX^™^ (Gibco, USA) supplemented with 10% fetal bovine serum (Gibco, USA) and antibiotic-antimycotic (Gibco, USA). Keratinocytes were cultured in collagen I-coated dishes (Corning, USA) using Epilife medium (Gibco, USA) with added S7 supplement (Gibco, USA). Green fluorescent protein-tagged HUVECs were purchased from Angioproteomie (USA). The HUVECs were cultured on collagen I-coated dishes (Corning, USA) using endothelial growth medium MV2 (Promocell, USA). Fibroblasts, A375, MeWo and HUVECs were detached using trypsin-EDTA 0.05% (Gibco, USA), keratinocytes were detached with Accutase (Gibco, USA).

### Generation of the melanoma spheroids

Spheroid co-cultures were generated in four configurations: MeWo cells with fibroblasts and HUVECs (MeWo w/ FBs), MeWo cells with HUVECs only (MeWo w/o FBs), A375 cells with fibroblasts and HUVECs (A375 w/ FBs), and A375 cells with HUVECs only (A375 w/o FBs). Cells were collected and mixed in a neutralized, salt-balanced rat tail collagen type I solution at 2 mg/ml (Sigma-Aldrich, USA) at a density of 500,000 cells/ml of each cell type. In a 6-well plate 20μl drops of the cell-collagen mixture were formed. After droplet formation an inverted 6-well plate was placed in the incubator for 10 min for the collagen to polymerize. After the droplet spheroid co-cultures solidified they were covered in DERM medium (DMEM + 10% FBS + 1% antibiotic-antimycotic + Ascorbic acid 0,5mM) and cultivated for two days before integration into the dermis of eSM-chip.

### Generation of the edgeless skin melanoma chip (eSM-chip) model

For eSM-chip integration, spheroids containing MeWo cells with fibroblasts and HUVECs are referred to as MeWo constructs, and those containing A375 cells with fibroblasts and HUVECs as A375 constructs throughout this article.

**Figure 1:**
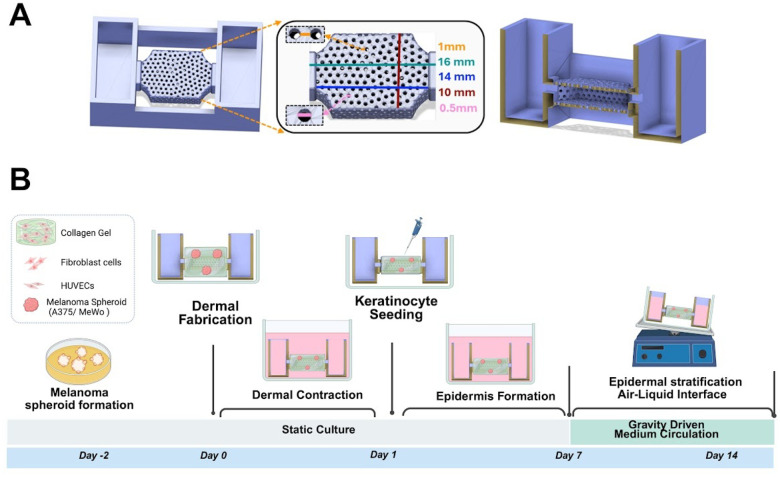
Scaffold design and eSM-chip development workflow. (A) Schematic of the 3D printed porous scaffold design and dimensions. A hollow porous scaffold is connected to two culture medium reservoirs, creating a pumpless system for gravity-driven perfusion. (B) Schematic depicting the workflow of generating eSM-chip, showing melanoma-containing spheroids embedded within a fibroblast-containing collagen dermal matrix, followed by epidermal layer formation and maturation. *Created with* Biorender.com

The 3D skin reconstruction protocol was adapted from Pappalardo et. al^13^ and modified for tumor spheroid and vasculature inclusion (Fig. 1). The 3D printed porous scaffolds (Fig. 1A) were coated with an 8% bovine gelatin type B (Sigma Aldrich, USA) and transglutaminase (400μg/ml in PBS) (Mark Nature, USA) using a fine paint brush and kept overnight in humid conditions at room temperature. Before coating, the gelatin was pasteurized at 75 C for 25 minutes, and the transglutaminase solution was filtered through a 0.22um syringe filter. The bottom of the device was sealed with a custom polydimethylsiloxane (PDMS) plug, forming a central well with the porous scaffold positioned in the middle.

Fibroblasts were collected and resuspended in a neutralized and salt balanced solution of collagen type I (3 mg/ml) at a density of 2,5 × 10^5^ cells/ml to form the dermal collagen mixture. The dermal mixture was poured in the central well and left at room temperature for five minutes for collagen to start polymerizing. After that the tumor spheroids were gently placed on the top of the dermal mix, after which the constructs were incubated at 37°C for 20 minutes to allow for full collagen polymerization. After full polymerization of the collagen plug was removed and the devices were submerged in DERM medium. The dermis was cultured for 24 hours before seeding keratinocytes on top of the reconstructed dermis. KCs were harvested at 90% confluence using accutase, resuspended in epidermalization (EPI) medium^15^ at 6 × 10^6^ cells/ml, and deposited on the surface of the dermis. Reconstructed dermis at the bottom and subsequently the top of the device was seeded with 100 μl of keratinocyte and incubated for 30 minutes at 37°C. Then, the culture vessel was filled with enough EPI medium to completely cover the edgeless skin constructs, which were cultured submerged in the medium for six days. The medium was changed every other day. On day seven, the devices were transitioned to ALI culture by removing culture medium from around the scaffold, and adding 2.5 ml of cornification (CORN)^15^ medium in the device reservoirs. During ALI the devices were cultured on a rocking platform, with 15° maximum incline and frequency of 0.13 Hz.

For vascularized constructs, HUVECs were incorporated at a concentration of 1×10^6^ cells/mL combined with fibroblasts and cultured in serum-free medium consisting of DMEM/F12 supplemented with 1% BSA, 1X Insulin-Transferrin-Selenium, 1% Antibiotic-Antimycotic, 0.1 nM T3, 100 μg/mL ascorbic acid, 0.2 μg/mL hydrocortisone, 10 ng/mL VEGF, and 1 ng/mL FGF-2, adapted from^16^. Constructs were maintained in submerged culture for up to one week.

### Histochemical staining

Reconstructed tissue was cut off of the scaffolds after culturing, washed with PBS and fixed in 4% paraformaldehyde in PBS overnight at 4°C. After fixation samples were divided in half – one half was cryopreserved in OCT, the other embedded in paraffin. The haematoxilin - eosin staining was performed following standard H&E protocol. The picrosirius red staining was done following the manufacturer’s instructions (VitroVivo Biotech, USA). For both stainings 10 μm tissue sections were used. Images were acquired with Leica SCN400 (Leica, Germany)

### Immunofluorescent staining

Cryopreserved tissue samples were cryosectioned (16 μm) and sections were allowed to dry overnight at room temperature. Sections were then permeabilized with 0.1% Triton X for 10 minutes, followed by blocking in PBS containing 8% bovine serum albumin (BSA) and 5% donkey serum. Primary antibodies diluted in blocking solution were added and the sections were incubated overnight (> 12 h) at 4°C. The following primary antibodies were used: keratin 14 (BioLegend, USA), α-smooth muscle actin (Cell Signaling Technology, USA), Ki67 (Abcam, UK), cleaved caspase-3 (Cell Signaling Technology, USA), MLANA (GeneTex, USA), and FAP (Cell Signaling Technology, USA). Alexa Fluor Plus donkey secondary antibodies (Invitrogen, USA) were applied for detection of primary antibodies and diluted in PBS containing 8% BSA. Cell nuclei were counterstained with DAPI. At a portion of experiments tumor mass was labeled by mixing in fluorescent carboxylate microspheres (Polysciences, USA). Fluorescence images were acquired using a Stellaris 5 confocal microscope (Leica Microsystems, Germany).

### Image analysis and quantification

Fluorescence images were acquired as 16-bit TIFF files, and z-stacks were maximum-intensity projected prior to analysis. Unless otherwise specified, image analysis was performed in QuPath (v0.4+), with nuclei detected using the DAPI channel.

Cleaved caspase-3 (Casp3) mean fluorescence intensity (MFI) was quantified within the defined tumor region and expressed as fold change relative to the control group mean (control = 1). For the MeWo construct, melanoma cells were identified within manually annotated dermis regions using per-cell detection of the MLANA channel, and Casp3 MFI was measured in the cytoplasm of MLANA-positive cells. For the A375 construct, the tumor region was defined by the ECM bead fluorescence channel and Casp3 MFI measured across all detected cells within this region.

For Ki67 proliferation index analysis in the MeWo construct, nuclei within annotated tumor regions were classified as MLANA-positive melanoma cells or non-melanoma cells using a trained object classifier in QuPath. Ki67 positivity was determined using a per-image internal reference threshold, defined as the mean + 2.5 SD of nuclear Ki67 fluorescence intensity in manually annotated quiescent dermal fibroblasts^17,18^. The Ki67 proliferation index was calculated as the percentage of MLANA-positive/Ki67-positive cells among all MLANA-positive cells within each tumor region. For the A375 model, the tumor region was defined using the ECM bead fluorescence channel. Ki67 proliferation was quantified as the percentage of cells within this area classified as Ki67-positive, based on nuclear Ki67 intensity measured with a Python analysis pipeline. For each image, two candidate thresholds were generated from per-nucleus Ki67 intensity: the 95th percentile of Ki67 intensity in manually annotated quiescent dermal fibroblasts, used as an internal reference threshold, and a Gaussian mixture model fit to the tumor Ki67 distribution^19^, used when the distribution was bimodal. The final threshold was selected for each image by reviewing the Ki67/DAPI overlay together with these two reference values.

For the MeWo construct, FAP and αSMA expression were quantified per cell within annotated tumor and dermal regions. FAP was measured across detected cells, whereas MLANA-positive melanoma cells were excluded from αSMA analysis. For the A375 construct, FAP and αSMA expression were quantified per cell across detected cells within defined tumor and dermal regions. Cell distance to the nearest tumor boundary was measured from each cell centroid and used to assign cells to intratumoral, proximal stromal (0–100 μm), or distal stromal (> 100 μm) regions. FAP values were normalized to tumor-free gel controls for each model, whereas αSMA values were normalized to distal stromal regions located > 300 μm from the tumor boundary.

Vascular networks were quantified from maximum-intensity projections of HUVEC-GFP images. Images were binarized, and skeletonized in Fiji^20^. Vessel thickness was measured using the Local Thickness plugin^21^, and vessel area fraction (%) was calculated as the fraction of the image occupied by the binarized vessel mask. Four non-overlapping 500 μm concentric annular zones were generated outward from the tumor boundary. Within each zone, skeletonized images were analyzed using the AnalyzeSkeleton plugin^22^ to quantify vessel length density (VLD, μm/μm^2^), average branch length (μm), and branch density (/mm^2^). All zone-based metrics were normalized to the tissue-clipped area of each zone. For tumor spheroid co-cultures, vascular metrics were quantified across the full image area.

All image analysis pipelines were developed by the authors, with computational assistance from Claude (Claude Sonnet, Anthropic). All analytical decisions and outputs were critically evaluated and validated by the authors.

### Statistics

Statistical analyses and data visualization were performed using BioRender Graphing (BioRender, Toronto, ON, Canada; RRID), powered by R version 4.5.1.. Data are shown as box plots with the median, interquartile range, minimum-to-maximum whiskers, and individual data points. All tests were two-tailed, and statistical significance was defined as α = 0.05. Exact p-values are reported for significant comparisons.

Normality was assessed using the Shapiro–Wilk test implemented within BioRender Graphing prior to statistical test selection. For normally distributed datasets, comparisons across three or more groups were performed using one-way ANOVA with Tukey or Bonferroni post hoc correction, as appropriate. For non-normally distributed datasets, Kruskal–Wallis testing with Dunn’s post hoc correction was used. Conditions with n = 2 were not subjected to inferential statistical testing and are presented descriptively.

## Results

### Tumor spheroids integrate into the eSM-chip and establish their microenvironment

**Figure 2:**
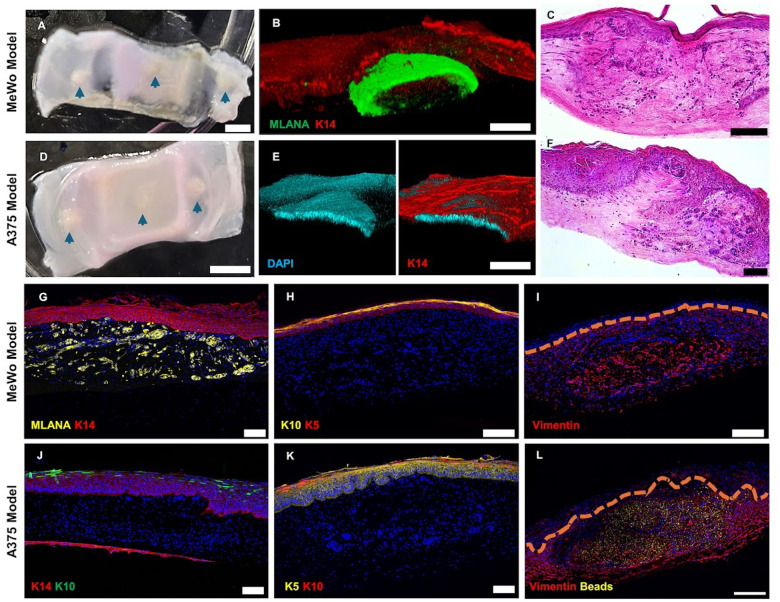
The eSM-chip exhibits spatial tumor spheroid integration and organized epidermal-dermal architecture. (A,D) Macroscopic appearance of the edgeless human skin melanoma equivalents; (B,E) Whole-mount immunostained 3D confocal images confirm tumor spheroid integration and epidermal keratin 14 (K14) expression (Scale bar: 250μm). (C, F) H&E-stained sections reveal distinct dermal and epidermal layers with an integrated tumor spheroid (Scale bar: 250μm); (G-L) cross-sections demonstrate epidermal and dermal marker organization in the eSM-chip. Scale bar: 5mm (A, D); 250μm (B,E,G-L)

We incorporated spheroids containing MeWo or A375 cell lines into the eSM-chip. Reconstructed melanoma tumors were macroscopically visible for both cell lines (Fig. 2A and D; arrows). Whole-mount immunofluorescent (IF) staining and confocal imaging (Fig. 2B and E) further confirmed tumor localization in the eSM-chip. In MeWo constructs, MLANA staining outlined the tumor mass beneath the K14-positive reconstructed epidermis (Fig. 2B), indicating correct anatomical placement. A375 cells are known to exhibit a highly dedifferentiated phenotype and did not show positive immunostaining to neither MLANA nor S100B. This is supported by the fact that MLANA and S100B are downstream proteins of MITF which expressed at low levels in A375^23^, therefore the tumor mass was identified morphologically as a dense, spherical cell cluster identified by DAPI stained nuclei located directly below the K14-positive epidermal layer (Fig. 2E).

Histological H&E-stained sections (Figs. 2C and F) demonstrated that both MeWo and A375 derived tumors form heterogeneous multicellular masses within the reconstructed skin, consistent with clonal tumor outgrowth in a physiologically relevant tissue context. In both models, the tumor was well integrated into the dermal layer. IF staining of cryosections of the melanoma constructs showed tumor positioning along the basement membrane and within the dermis (Figs. 2G and L), recapitulating dermal invasion observed in human melanoma. We further confirmed the epidermal stratification with localized K10 and K14 expression (Figs. 2H and K), as well as the Vimentin-positive dermal fibroblasts surrounding the tumor cells (Figs. 2I and L).

**Figure 3:**
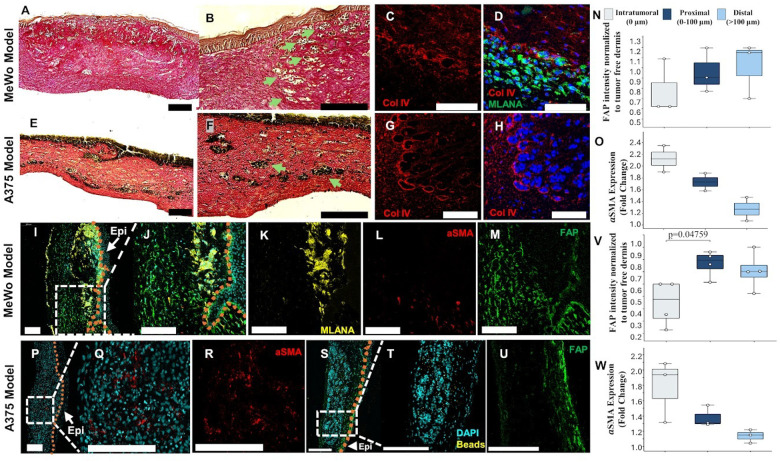
Reconstructed tumor spheroids exhibit peripheral metastatic cyst-like formations and CAF induction (A-B; E-F) Picrosirius red staining reveals difference in collagen fiber organization between the tumor spheroids and surrounding dermis, with non-polarized collagen in the tumor regions and polarized collagen in the dermal compartment. Metastatic cyst-like formations are observed at the periphery of the tumor spheroids, indicated by arrows; (C-D; G-H) Collagen IV staining marks metastatic cyst-like formations at the periphery of the tumor spheroids; (I-W) Cancer associated fibroblasts observed in close proximity to the tumor spheroid in both MeWo model (I–M and V-W) and A375 model (P-U and N-O). Scale bars: 200μm (A-B; F-G; J-N); 100μm (C-D; H-I). FAP and αSMA expressions were quantified in the MeWo and A375 model. FAP: MeWo Model, one-way ANOVA with Bonferroni correction, n=4 independent tumors; A375 model, Kruskal-Wallis test with Dunn’s post-hoc comparison, n=3 independent tumors. αSMA: MeWo Model, one-way ANOVA with Bonferroni correction, n=3 independent tumors; A375 model, descriptive analysis only, n=2 independent tumors.

Next, we characterized the cellular and extracellular microenvironment proximal and distal to the tumor. Picrosirius red staining of melanoma constructs (Fig. 3A, B, E, and F) revealed distinct ECM organization between the tumor compartment and the surrounding reconstructed dermis for both MeWo and A375.

Collagen fibers within the tumor compartment appeared disorganized and non-polarized, in contrast to the aligned architecture of the dermal ECM. Within the tumor compartment, cyst-like metastatic structures were observed (Figs. 3B and F; arrows), with increased frequency toward the tumor periphery and the basement membrane zone. Portions of the metastatic cyst-like structures were enclosed within a Collagen IV-positive membrane (Figs. 3C, D, G and H). A375, derived from a skin metastatic lesion^24^ and MeWo, established from a lymph node metastasis^25,26^, this morphology is consistent with retained metastatic potential and the ability to generate secondary tumor foci within the engineered tissue context. In addition, CAF marker FAP was enriched in the tumor periphery, both for MeWo (Figs. 3M–V) and A375 (Figs. 3U–N), compared to intra-tumoral expression, whereas αSMA, another CAF marker, decreased with increasing distance from the tumors of both cell lines, suggesting microenvironmental reprogramming of dermal fibroblasts into a CAF-like state with differential activation of CAF markers with respect to tumor proximity (Figs. 3L, O, R and W).

### Vascular formation in the eSM-chip depends on the presence of fibroblasts, tumor cell line, and tumor proximity

**Figure 4:**
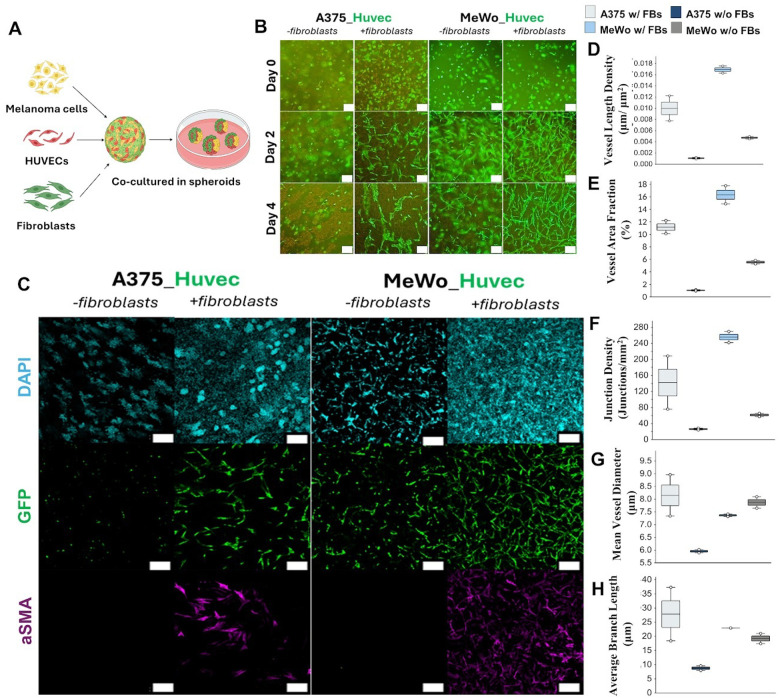
Vascular network formation is enhanced by fibroblast incorporation and varies between A375 and MeWo eSM-chips. (A) Schematic overview of tumor spheroid generation for vascularized eSM-chip models. (B) Representative images captured from day 0 to day 4 showing vascular network formation in A375 and MeWo models with or without fibroblasts. (C) Representative fluorescence images showing nuclei (DAPI), endothelial networks (GFP), and αSMA expression across the different eSM-chip conditions. (D) Quantification of vascular network parameters, including vessel area fraction (%), vessel length density (VLD, μm/μm^2^), mean vessel diameter (μm), average branch length (μm), and junction density (/mm^2^). Fibroblast incorporation increased vascular network metrics, while the MeWo model showed higher vessel area fraction, VLD, and junction density compared to A375 model. Mean vessel diameter and average branch length did not follow this trend. Vascular metrics were quantified across the full image area; n = 2 independent tumor spheroids per condition. Data are shown descriptively; no statistical analysis was performed.

After establishing the eSM-chip, we aimed to integrate it with a microvascular network using GFP-tagged HUVECs. To match the initial cellular composition of the tumor spheroids with the surrounding stroma, we began with vascularizing tumor spheroids (Fig. 4A).

Fibroblast incorporation into the cell-collagen mixture visibly enhanced HUVEC maintenance and promoted more pronounced vascular network formation within tumor spheroids over time (Figs. 4B and C). In A375 spheroids, baseline vasculature was near absent, with vessel area fraction and vessel length density increasing approximately 9–10 fold upon fibroblast co-culture (Fig. 4D and E). In MeWo spheroids, fibroblasts further enhance an already detectable baseline network, increasing vessel area fraction and vessel length density by 3–4 fold (Fig. 4D and E). Vascular network architecture also differed between cell lines. Compared to A375, MeWo spheroids showed 45% greater vessel area coverage, 70% higher vessel length density, 80% enhanced junction density, 18% shorter average branch length, and 10% reduced mean vessel thickness when integrated into (Fig. 4D–H).

When tumor spheroids were integrated into the vascularized eSM-chip, overall MeWo constructs exhibited 2.35-fold higher vessel area fraction, 3.65-fold higher vessel length density, and approximately 15-fold higher junction density compared to A375 constructs (Supplementary Fig. 1A-D).

**Figure 5:**
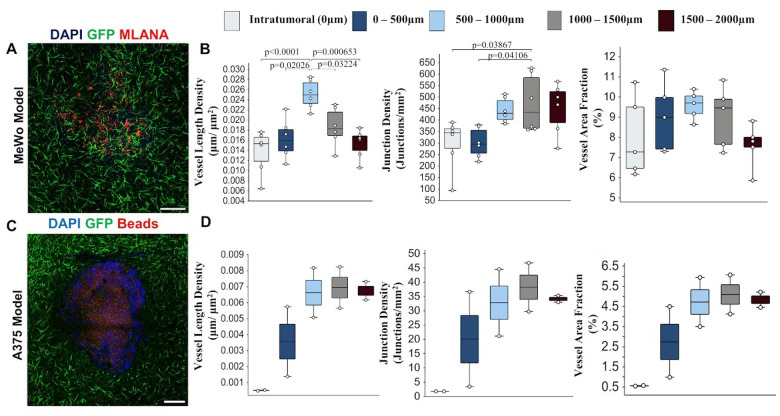
Integrated vascularized tumor spheroids show spatially patterned vascular organization within the vascularized dermal compartment. (A) Representative fluorescence images showing nuclei (DAPI), endothelial networks (GFP), and MeWo cells labelled by MLANA within the vascularized model. (B) Spatial zone-based quantification of vascular network parameters around the integrated MeWo tumor spheroid, including vessel area fraction (%), vessel length density (VLD, μm/μm^2^), mean vessel diameter (μm), average branch length (μm), and junction density (/mm^2^). Metrics were quantified across consecutive 500 μm zones and normalized to the tissue-clipped area of each zone. (C) Representative fluorescence images showing nuclei (DAPI), endothelial networks (GFP), and ECM beads marking the integrated A375 tumor spheroid region within the vascularized dermal compartment. (D) Similar spatial zone-based quantification of vascular network parameters around the integrated A375 tumor spheroid model. For the MeWo model, one-way ANOVA with Tukey HSD post-hoc correction was performed; n = 6 independent vascularized tumor models for vascular length and junction density, and n = 5 for vessel area fraction. Exact p-values are given in the graphs. For the A375 model, data are descriptive only; n = 2 independent vascularized tumor models, with no statistical comparison performed.

Spatially, the intratumoral region of MeWo constructs showed 7.3% vessel area fraction and 356 junctions/mm^2^, while A375 constructs were largely avascular intratumorally, with vessel area fraction of 0.5% and junction density of 1.7/mm^2^. With increasing distance from the tumor spheroid, MeWo constructs showed moderate increases across all metrics, peaking at 500–1000 μm with 1.1–1.3 fold changes before declining back toward intratumoral levels by 1500–2000 μm (Fig. 5A). In contrast, A375 constructs showed a marked increase from the avascular intratumoral region, with vessel length density, vessel area fraction, and junction density increasing approximately 14-fold, 9-fold, and 20–22 fold respectively by the 1000–1500 μm ring, followed by a modest decline at 1500–2000 μm (Fig. 5B). This data indicates distinct angiogenic behavior between melanoma backgrounds, with A375 vascularity being largely confined to the distal stroma, whereas MeWo maintained intratumoral and stromal vascularity.

### eSM-chip responds to targeted therapy with BRAF and MEK inhibitors

We next evaluated the ability of the eSM-chip to capture tumor response to targeted therapy. A375 tumors, which harbor the BRAF V600E mutation, were treated with the BRAF inhibitor PLX4720 at two concentrations (5 μM and 40 μM). Treatment was initiated at the air-liquid interface on day 1 and maintained for 7 days via supplementation of the culture medium.

**Figure 6:**
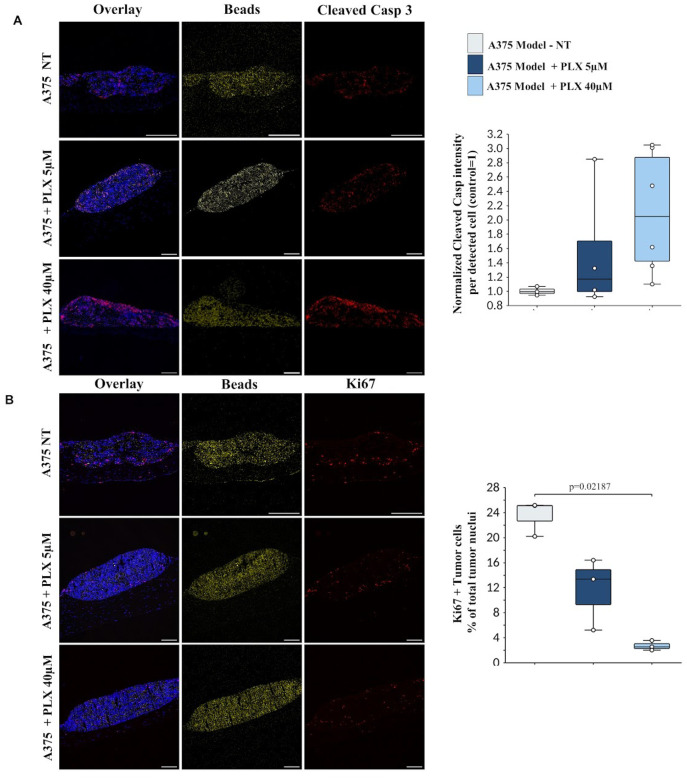
PLX-mediated BRAF inhibition increases caspase-3-associated apoptosis and reduces Ki67-positive proliferating cells in the A375 model. (A) Representative fluorescence images showing overlay, ECM beads and cleaved caspase-3 staining in untreated, 5 μM PLX-treated, and 40 μM PLX-treated A375 models. Quantifications show dose-dependent changes in cleaved caspase-3 mean fluorescence intensity (MFI) within the tumor spheroid, measured per cell and normalized to the untreated control group. (B) Representative fluorescence images showing overlay, ECM beads and Ki67 staining in untreated, 5 μM PLX-treated, and 40 μM PLX-treated A375 models. The proliferation index is shown as the percentage of Ki67-positive tumor nuclei among total tumor nuclei counted within tumor spheroid. Scale bar: 200 μm. For cleaved caspase-3, one-way ANOVA with Tukey post-hoc correction was performed; control n = 3, 5 μM PLX n = 4, and 40 μM PLX n = 6 independent tumor spheroids. For Ki67, Kruskal–Wallis test with Dunn’s post-hoc correction was performed; control n = 3, 5 μM PLX n = 3, and 40 μM PLX n = 3 independent tumor spheroids. Exact p-values are indicated on the graphs.

Treatment with PLX4720 resulted in a concentration-dependent increase in cleaved caspase-3 signaling with 5 μM and 40 μM, PLX4720 producing approximately 1.55-fold and 2.10- fold increases, respectively, compared with untreated A375 model (Figs. 6A). Ki67-positive cells within the tumor region decreased from 23.5% in untreated constructs to 11.6% at 5 μM and 2.7% at 40 μM (Figs. 6B). Together, these results indicate elevated apoptosis and reduced proliferation in response to BRAF inhibition (Figs. 6B).

**Figure 7:**
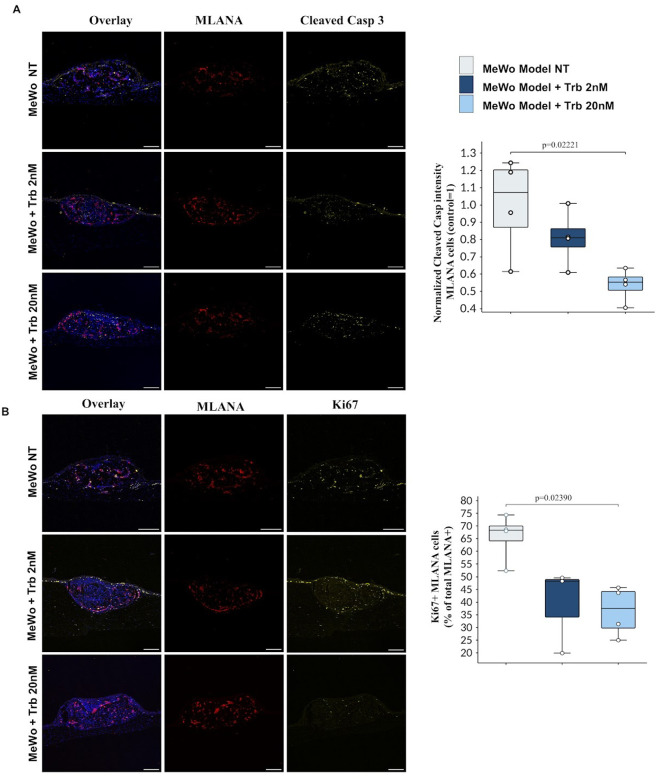
Trametinib-mediated MEK inhibition reduces cleaved caspase-3 signal and Ki67-positive proliferation in the MeWo model. (A) Representative fluorescence images showing overlay, MLANA-positive MeWo cells, and cleaved caspase-3 staining in untreated, 2 nM and 20 nM Trametinib- treated MeWo models. Quantification shows cleaved caspase-3 mean fluorescence intensity (MFI) within MLANA-positive tumor cells, expressed as fold change relative to the untreated control. (B) Representative fluorescence images showing overlay, MLANA-positive MeWo cells, and Ki67 staining in untreated, 2 nM and 20 nM Trametinib-treated MeWo models. The proliferation index is given as the percentage of Ki67-positive cells among total MLANA-positive tumor cells counted. Scale bar: 200 μm. For cleaved caspase-3, one-way ANOVA with Bonferroni post-hoc correction was performed; n = 4 independent tumor spheroids per group. For Ki67, one-way ANOVA with Bonferroni post-hoc correction was performed; control n = 4, 2 nM trametinib n = 3, and 20 nM trametinib n = 4 independent tumor spheroids. Exact p-values are indicated on the graphs.

MeWo constructs were treated with the MEK inhibitor trametinib at 2 nM or 20 nM using the same ALI-based approach. Unlike A375, caspase-3 mean activity decreased with increasing trametinib concentration, showing approximately 7% and 41% lower activity at 2 nM and 20 nM, respectively, compared with untreated MeWo model (Fig. 7A). Ki67-positive cells within the tumor region decreased from 65.8% in untreated constructs to 39.2% and 36.4% at 2 nM and 20 nM respectively, indicating reduced proliferation following MEK inhibition (Fig. 7B).

## Discussion

We established the eSM-chip as a melanoma-affected skin model that recapitulates key structural and microenvironmental features of *in situ* melanoma. Our strategy allows for melanoma spheroids (A375 and MeWo) to localize beneath the reconstructed K14-positive epidermis, along the basement membrane zone, and integrate into the reconstructed dermis with a dermal invasion-like topology. This spatial organization resembles late vertical growth phase melanoma, where tumor expansion progresses below the epidermal–dermal interface with a deep dermal invasion. Histological and whole-mount analyses confirm that tumor spheroids are structurally integrated with the dermis rather than superficially attached, supporting the structural fidelity of the model.

Mechanical boundary conditions, such as ECM organization and stiffness, are not neutral variables in tumor modeling as they actively regulate melanoma cytoskeletal remodeling and transcriptional programs that promote invasion and metastasis^2,14,27,28^. In this study, we chose to use the edgeless skin reconstruction strategy as our previous work established that the edgeless approach significantly enhances ECM deposition, dermal cell and ECM organization, and mechanical properties compared to conventional 3D skin constructs that have open lateral edges^12,13^. Consistent with this concept, we observed compartment-specific ECM organization within the melanoma constructs. Picrosirius red staining demonstrated disorganized collagen architecture within the tumor compartment compared to the aligned fibers of the surrounding reconstructed dermis. Moreover, collagen IV-positive cyst-like structures at the tumor-stroma boundary suggest localized remodeling of the basement membrane region, a hallmark of invasive melanoma progression^29,30^.

αSMA and FAP exhibited inverse spatial expression within the tumor microenvironment, with intratumoral enrichment of αSMA and greater FAP expression in more distal dermal regions. Notably, FAP expression showed tumor cell line-dependent variation, with significant proximal enrichment in MeWo and a non-significant increase across proximal and distal regions in the A375 construct. Together, these findings raise the possibility that fibroblast activation in our model is spatially organized and may reflect distinct CAF activation states. This is in line with the role of CAFs in melanoma invasion, matrix remodeling, and therapeutic resistance^31^, as well as organotypic melanoma-in-skin studies showing that fibroblast activation and ECM remodeling shape tumor behavior^9^. Given the heterogeneity of CAF phenotypes^31,32^, the intratumoral enrichment of αSMA is consistent with a myofibroblastic CAF-like state (myCAF)^31^ although whether this activation is driven by biochemical and/or biomechanical signaling remains to be determined. The greater FAP expression observed in distal dermal regions further suggests that the dermal fibroblasts in eSM-chip are still in an activated, ECM-remodeling state, as opposed to a quiescent state, and their activation could be dampened by the tumor signals. Although requiring further investigation, this data is supportive of the CAF heterogeneities previously described for melanoma^33,34^.

Our vascularization studies further highlight the importance of stromal composition, in this case fibroblast presence, in microenvironment engineering. The incorporation of fibroblasts within melanoma spheroids enhanced microvascular network formation. In the spheroid experiments it could be seen that vessel length and junction density, area fraction and longevity were improved with fibroblast presence. The pro-angiogenic and vessel-stabilizing roles of fibroblasts have been described in both tumor and non-tumor engineered tissues^35,36^, where fibroblast-derived growth factors (e.g., VEGF, FGF, PDGF) and matrix remodeling enzymes support endothelial survival and lumen formation^37^. Microvascularized skin equivalents and melanoma-in-skin systems have demonstrated that stromal-vascular interactions critically influence tumor growth and drug response^8^. In tumor spheroid co-cultures, MeWo cells appeared to form denser and more interconnected vascular networks with higher vessel area fraction, vessel length density, and junction density, whereas A375 was associated with greater average branch length. Constitutive MAPK signaling downstream of BRAF V600E in A375 cells may contribute to this phenotype by promoting tumor cell proliferation and invasion^38,39^. A more proliferative tumor mass could limit HUVEC network formation through increased cellular crowding and metabolic demand, particularly given that proliferating endothelial cells are highly dependent on glucose and glutamine metabolism^40^. Together, reduced vascularization in A375 construct may reflect combined physical and metabolic constraints, although the relevant cellular and metabolic parameters remain to be directly measured.

When tumor spheroids were subsequently integrated into the vascularized eSM-chip, the same vascular trends were observed, with MeWo spheroids associated with denser and more interconnected vascular networks compared to A375, suggesting tumor-intrinsic differences in HUVEC self-organization. Vascular networks were transient in both systems, regressing after approximately one week. Despite the presence of stromal support from fibroblasts, this regression may be due in part to the absence of perfusion in the current model configuration. Integration into a microfluidic platform with controlled perfusion represents a logical next step, as shear stress has been shown to promote endothelial maturation, barrier formation, and vessel stabilization, enabling more physiologically relevant modeling of tumor-vascular interactions and immune trafficking^41^. Such functionalization would open avenues for investigating immunotherapeutic response, metastatic dissemination, and clonal cancer stem cell dynamics under dynamic flow conditions.

In A375 constructs, PLX4720 treatment increased cleaved caspase-3 and reduced Ki67 in a concentration-dependent manner, consistent with clinically-established apoptotic cell death and suppression of proliferation following BRAF inhibition. However, because BRAF inhibition can also induce therapy-associated senescence in BRAF-mutant melanoma^42^, a subset of growth-arrested but viable cells may coexist with the apoptotic population. In MeWo constructs, trametinib reduced Ki67 and decreased cleaved caspase-3 levels below baseline control in a concentration-dependent manner, suggesting reduced proliferation without a parallel increase in apoptosis. This aligns with reports that MEK inhibition can suppress melanoma cell growth through G1 arrest without necessarily inducing apoptotic cell death^43^. Furthermore, in BRAF wild-type melanoma cells, MEK inhibition has also been linked to compensatory PI3K/AKT survival signaling, which may help explain the reduced caspase-3 activity observed here^43^.

Together, eSM-chip provides an advanced melanoma-affected skin model that incorporates tumor, epidermal, stromal, and vascular compartments, capable of capturing multicellular interactions that cannot be recapitulated in melanoma cell cultures alone. The findings on vascular network formation, stromal activation, and mutation dependent drug responses support the use of the eSM-chip as a controlled human tissue platform for investigating melanoma–microenvironment crosstalk and therapeutic responses.

## Data Availability

The authors declare that the data supporting the findings of this study are available within the paper and its Supporting Information. Should any raw data files be needed in another format, they are available from the corresponding author upon reasonable request.
